# Moral Foundations Predict Religious Orientations in New Zealand

**DOI:** 10.1371/journal.pone.0080224

**Published:** 2013-12-10

**Authors:** Joseph Bulbulia, Danny Osborne, Chris G. Sibley

**Affiliations:** 1 Victoria University of New Zealand, Wellington, New Zealand; 2 Department of Psychology, University of Auckland, Auckland, New Zealand; University of Utah, United States of America

## Abstract

The interplay between religion, morality, and community-making is a core theme across human experience, yet scholars have only recently begun to quantify these links. Drawing on a sample of 1512 self-identified religious – mainly Christian (86.0%) – New Zealanders, we used structural equation modeling to test hypothesized associations between Religious Orientations (Quest, Intrinsic, Extrinsic Personal, Extrinsic Social) and Moral Foundations (Care/Harm, Fairness/Cheating, Loyalty/Betrayal, Authority/Subversion, Sanctity/Degradation). Our results show, for the first time in a comprehensive model, how different ways of valuing communities are associated with different ways of valuing religion.

## Introduction

Religious Orientations theories (RO) and Moral Foundations theory (MFT) offer distinct theoretical perspectives in the moral psychology of religion. We tested hypothetical associations between RO and MFT constructs in a large population of self-identified religious adherents in New Zealand. Investigating these associations is important for three reasons.

First, RO and MFT are influential theoretical positions in the moral psychology of religion. Yet no previous study has assessed how their central constructs relate. This study addresses that gap.

Second, MFT researchers have challenged the empirical adequacy of RO constructs. Specifically, MFT researchers have worried that RO too narrowly focuses on individual faith without sufficiently assessing faith's social dimensions [1] p.142. However, such challenges have been mounted on theoretical grounds. By assessing predicted associations between MFT and RO, we empirically evaluate each tradition in terms of the other's central constructs.

Third, little is known about the relationship between religious and moral cognition. Investigating how ways of valuing communities relate to ways of valuing religion is an important step forward in the larger project of explaining how religion and community-making affect each other [2]–[3].

### Religious Orientations

Religious orientations research began in the 1950s from efforts to understand the relationship between religion and prejudice. Early RO researchers were puzzled by evidence that strongly religious people exhibit more prejudice than do weakly religious and non-religious people [4]. To early RO researchers, such prejudice appeared to be at odds with religious doctrines of equality and universal love [4]. Allport and Ross (1967) noticed that even if a majority of church-goers were prejudiced, a substantial minority exhibited less-than-average levels of prejudice. The authors conjectured that religious individuals differ in their social attitudes, some internalizing religion more than others, and that such differences can be predicted from their attitudes to faith. Allport and Ross posited a continuum of orientations to faith; at the extrinsic end of this continuum are those who follow religion for personal ends, “Extrinsic values are always instrumental and utilitarian” [5] p.424; at the intrinsic end are those who “find their master motive in religion” [5] p.434. Despite its initial appeal, the idea that religious orientations could vary on a single continuum was later found to be inadequate for capturing the diversity of religious orientations. For example, some religious people appear to follow religion as a master motive, yet also conceive of faith as a journey directed to some non-specified end: religion as “quest” [6]. In place of a continuum, RO researchers eventually developed a four-fold categorical typology:

1. **Quest Orientation** regards religion as a spiritual journey that permits both doubt and discovery about faith [7]. Quest conceives of religion as a search rather than a destination. Notably, Quest is associated with lower prejudice [8].
2. **Personal Extrinsic Orientation** values faith as a means to individual benefit, such as personal comfort or good health [9]. Religion is valued for what it brings to the religious person.
3. **Social Extrinsic Orientation** values faith as a means to interpersonal connection, such as interacting with friends and meeting new people [10]. Religion is valuable for what it brings to a community of religious people.
4. **Intrinsic Orientation** regards religion as a way of life that is good in itself, rather than as a journey or as a means for obtaining ulterior benefits [11]. Those who score high on intrinsic religion see religion as right and true, irrespective of any personal or social benefit, and irrespective of any journey of personal discovery – a “master motive”. Notably, intrinsics appear to be highly sensitive to religious cues when making moral judgments, and place a high value on norms that are associated with religion [12] p.323.

### Moral Foundations Theory

Moral Foundations Theory attempts to accommodate moral psychology within a broader, life science perspective on human social psychology. According to MFT, morality evolved as a functional adaptation for building smaller and larger-order social groups [13]. In humans, genetic endowment provides a “first draft” moral mind which is also responsive to cultural learning, supporting culturally diverse moralities [13] p.63. MFT describes two broad dimensions of moral judgment. *Individualizing foundations* pertain to the treatment of persons, their rights and protections. *Binding foundations* pertain to the treatment of communities, what is owed to the groups as such [13]. Within individual and binding foundations, MFT describes a total of five further subcategories. Somewhat confusingly, MFT also refers to these sub-categories as Foundations, describing two individual foundations – (1) Care/Harm, (2) Fairness/Cheating – and three binding foundations (3) Loyalty/Betrayal, (4) Authority/Subversion, (5) Sanctity/Degradation. We next describe MFT's five moral foundations and introduce our hypothesized links between MFT and RO.

1. **Care/Harm (Individualizing Foundation)** MFT holds that part of morality originates from the human capacity to nurture and protect – *Care*. “Whatever functional systems made it easy and automatic to connect perceptions of suffering with motivations to care, nurture, and protect are what we call the Care/Harm foundation” [13]. Notably, MFT holds that Care is a basic biological feature, which extends beyond humans to a diversity of lineages because “[a]ll mammals face the adaptive challenge of caring for vulnerable offspring for an extended period of time” [13] p.67. MFT also argues that Westerners tend to be more focused on the avoidance of Harm than non-Westerners who tend to be more focused on the Binding foundations [14] p.1001. Moreover MFT observes that political liberals tend to emphasize Care more than the Binding Foundations of Authority, Loyalty, and Sanctity when compared to political conservatives [15] or libertarians [16]. Might differences in Care track differences in religious orientations? Because RO does not suggest differences for religious orientations and Care, we did not predict a significant association between Care and religious orientations (Prediction 1).
2. **Fairness/Cheating (Individualizing Foundation)** MFT holds that moral judgment is partially grounded in a sense of what is justly owed to others – *Fairness*. MFT conjectures that Fairness evolved from the evolutionary demands of close interpersonal interactions based on reciprocity [13]. Notably, MFT has observed that Fairness varies with political ideology: political conservatives more strongly endorse Fairness than do liberals [17]. Might differences in Fairness track differences in religious orientations? RO research consistently finds a positive association between Quest orientation and support for the equal treatment of people irrespective of group membership [12] p.321. Based on these findings, we hypothesized that Fairness would be positively associated with Quest (Prediction 2).
3. **Loyalty/betrayal (Binding Foundation)** MFT argues that part of morality arises from commitments to abstract conceptions of community – *Loyalty*. According to MFT, Loyalty evolved for effective social coordination [13]. MFT also conjectures that religions evolved to enhance Loyalty: “[r]eligious narratives and teachings are often aimed at the creation and maintenance of a people, church, or nation, stressing the moral obligations of loyalty and self-sacrifice for this group above all other groups” [1]. Though MFT does not state whether Loyalty might be associated with differences in religious orientations, we hypothesized that Loyalty would be associated with both Individual and Social Extrinsic orientations. This is because strong religious communities have been linked with personal [18] and community-level benefits [19]–[20]. Based on MFT's hypothesis that Loyalty facilitates strong communities, we predicted that religious adherents who more strongly valued Loyalty would also tend to value religion for Personal and Social ends (Predictions 3a. and 3b).
4. **Authority/Subversion (Binding Foundation)** MFT holds that a moral psychology of Authority was conserved and amplified from enhancements to social coordination, and that religion underpins the psychology of Authority: “the world's major religions also include moral instruction in showing proper respect to authority figures, obeying rules and commandments, fulfilling the duties of one's social role, and respecting the traditions and institutions of the religious in-group” [1] p.143. MFT does not state whether differences in Authority might predict differences in religious orientations. However, based on RO research, we predicted two associations. First, we predicted that Authority would be negatively associated with Quest (Prediction 4a). This prediction was based on the idea that Quest emphasizes inner authority and openness to change, and that valuing traditional authority and valuing inner authority appear ostensibly opposed. Second, we predicted a negative relationship between Authority and Intrinsic orientation. This prediction was based on recent studies suggesting trade-offs between commitments to religious authority and commitments to secular authority [21]–[22]. Given that the MFT's Authority items do not imply religious authority, we predicted an inverse relationship between Authority and Intrinsic orientation (Prediction 4b).
5. **Sanctity/degradation (Binding Foundation)** MFT holds that part of morality is shaped by a psychology of disgust and contamination. Following Emile Durkheim's theory of religion as binding communities through sacred values, MFT posits that religious cultures support galvanizing moral responses by capitalizing on a psychology of disgust and contamination, linking this complex to moral judgments [1] p.1001. MFT's conception of a sanctity foundation accords with Tetlock's “Sacred Values Protection Model (SVPM),” which defines sacred values as those which “a moral community treats as possessing transcendental significance that precludes comparisons, trade-offs, or indeed any mingling with secular values” [23] p.320. In support of a contamination-avoidance Sanctity Foundation, it has been found that sacred values exhibit such intrinsic motivations [24] and that the violation of sacred values is associated with disgust and outrage [24]–[25]. Because Intrinsic orientations conceive of religion as inherently right and good, irrespective of any personal or social benefit, we predicted that Sanctity would be positively associated with Intrinsic orientations (Prediction 5).

## Results

### Structural Equation Model

We assessed concurrent associations between each moral foundation and religious orientation using Structural Equation Modeling (SEM). SEM improves on correlational analyses because associations between latent factors are formally estimated, thus adjusting for measurement error. Each latent factor was allowed to relate only to its specified manifest indicators (i.e., the individual items in that scale). Our SEM included only the hypothesized links between exogenous (Moral Foundations) and endogenous (Religious Orientations) latent factors. As such, our model assessed the significance of the formally hypothesized links between moral foundations and religious orientations, while adjusting for measurement error in these constructs. Our study did not test any formal causal model about how the latent factors we estimated are related. Rather, we tested associations between these latent factors. As indicated at the outset, assessing these associations is interesting because (i) there is currently no formal assessment of the links between RO and MFT; (ii) MFT researchers have questioned the relevance of RO on theoretical, not empirical, grounds; (iii) assessing relationships between the core constructs of MFT and RO is a basic precondition for constructing causal models.

**Figure 1:**
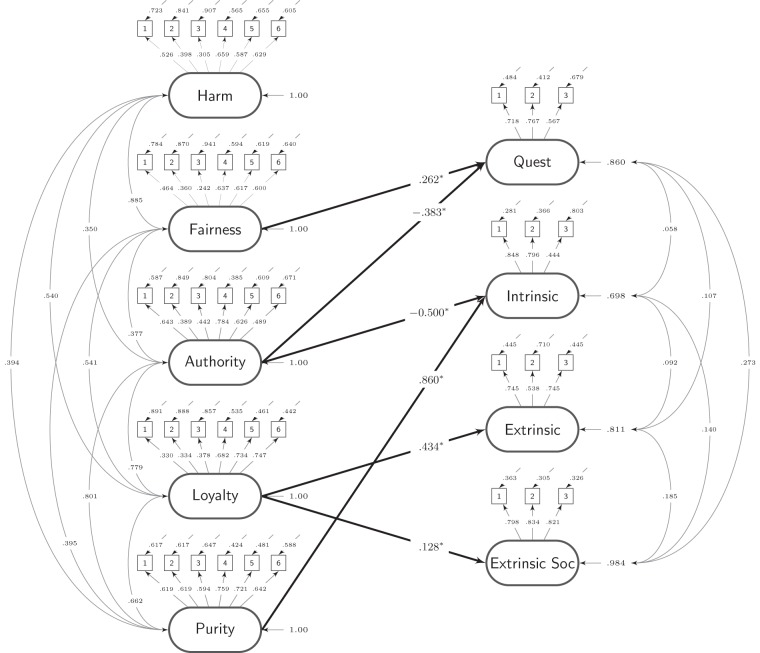
The hypothesized structural equation model with standardized parameters assessing the associations between moral foundations and religious orientations.

Given our large sample size, we adopted a conservative criterion for determining statistical significance: p<.001. Our SEM with standardized coefficients for all parameter estimates is presented in **Figure 1** **.**

For well-fitting models, the standardized Root Mean Square Residual (sRMR) should generally be below .080 and the Root Mean Square Error of Approximation (RMSEA) should generally be below .060 [26]. Based on these criteria, the hypothesized model presented in **Figure 1** provided a reasonable fit to the observed data, *x* ^2^ (797) = 3873.19, p<.001; sRMR  = .060; RMSEA  = .051; 90% CI for RMSEA  = .049,.052.

Consistent with past RO research showing that Quest is associated with greater support for equality, we found that Fairness was positively associated with Quest orientation, *β* = .262, se = .037, t = 7.043, p<.001.

As predicted, Loyalty was positively associated with Personal Extrinsic orientation (*β* = .434, se = .030, t = 14.528, p<.001) and Social Extrinsic orientation (*β* = .128, se = .032, t = 3.997, p<.001).

As predicted, Authority was negatively associated with Quest (*β* = −.383, se = .036, t =  −10.774, p<.001) and Intrinsic orientation (*β* =  −.500, se = .072, t =  −6.980, p<.001).

Finally, in line with past research suggesting a link between Sanctity and Intrinsic orientations, we found that Sanctity was positively associated with Intrinsic orientation (*β* = .860, se = .069, t = 12.527, p<.001). Against our theoretical model, we tested an alternative model in which the five moral foundations were allowed to related to each of the four religious orientations (*x* ^2^ (783)  = 3837.251, p<.001; sRMR  = .059, RMSEA  = .051; 90% CI for RMSEA  = .049,.052). This less-constrained model provided an improved model fit relative to the hypothesized model 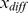 (14)  = 35.939, p = 0.001, however none of the additional unpredicted pathways reached significance (p's >0.001). We therefore opted for the hypothesized model, as it provides both a more parsimonious, as well as theoretically-derived, explanation for the data, with good relative fit, as compared to an alternative model including all links between moral foundations and religious orientations.

## Discussion

### Links Established between MFT and RO

RO and MFT are influential theoretical positions in the moral psychology of religion. Yet no previous study has assessed how their central measures relate. This study revealed, for the first time, how religious people who endorse certain moral foundations tend to identify with distinct religious orientations. Specifically, we observed the following associations:

1. **Care is unassociated with specific religious orientations (Prediction 1).** In accordance with the position of MFT that Care is a universal principle, we did not predict any relationships between Care and religious orientations. Indeed, in an unconstrained model including these non-predicted paths, we noted that they were all non-significant.
2. **Fairness is associated with Quest (Prediction 2).** Fairness has been found to vary with political orientations: liberals tend to endorse fairness more than do conservatives or libertarians [16]. Moreover, RO has consistently found that Quest is associated with greater support for equality [6], [12], [27]. We suggested that support for equality and the equal treatment of people is consistent with the Moral Foundation of Fairness; as such, it is not surprising that Fairness and Quest are related.
3. **Loyalty is positively associated with Personal Extrinsic orientation (Prediction 3a) and Social Extrinsic orientation (Prediction 3b)** Analyses supported the predicted associations between Loyalty and the Extrinsic Personal (3a) and Extrinsic Social (3b) Orientations. MFT posits that Loyalty facilitates strong coalitions, and orthogonal research has demonstrated both individual and social benefits from belonging to strong social groups. We suggested that those who place a higher value on Loyalty also tend to affiliate with religion for personal and social benefits. Notice that MFT helps clarify a lingering question from RO research. Early RO researchers report that high church attending Extrinsics tended to be particularly high in prejudice. Allport and Ross explained this effect as a failure to internalize religion's universal core message of acceptance and tolerance; however, this explanation has been criticized as lacking a clear theoretical basis [1], [28]–[29]. Give that those who value strong groups (Loyalty) also tend to value religion for extrinsic motivations, it is credible that prejudice is grounded in the personal and social benefits of religion that arise from external reinforcement contingencies owing to group membership. Whether those with higher extrinsic values exhibit more prejudice from a motive to protect such benefits is a matter for future investigations.
4. **Authority is negatively associated with Quest orientation (Prediction 4a), and also negatively associated with Intrinsic orientation (Prediction 4b)** Quest values faith as a journey, suggesting a reliance on individual learning and openness. We predicted that Authority would be negatively associated with Quest. Results supported this prediction. We also predicted that Authority would be negatively associated with Intrinsic orientation. Previous research has found that more confidence for secular authority is associated with less confidence for religious authority and vice versa, which is to say that religious and secular authority exhibit “hydraulic” effects [21]. Notably, scale items assessing Authority neither mention, nor imply, religious authority. In New Zealand's context of low institutional religious affiliation, we predicted that religious affiliates who scored higher on Authority would tend to score lower on Intrinsic religiosity. That we found such a relationship suggests there might be within-religion differences in hydraulic responses. It would seem important to focus on such differences within the larger category of religious adherents, a matter for future research.
5. **Sanctity is positively associated with Intrinsic orientation (Prediction 5)** We predicted that Sanctity would be positively associated with Intrinsic orientations. This is precisely what we found. Indeed, the strongest of all our observed relationships was between Sanctity and Intrinsic orientations. The strength of this relationship (i.e., β = .86) suggests that among those who affiliate with religion, Sanctity and Intrinsic orientation are nearly indistinguishable. This suggests a use for RO in promoting a more sophisticated moral psychology of religion than MFT currently offers. MFT points out that studies based on North American Protestants offer limited inferential power. Future research must consider whether Sanctity foundations, in the context of religion, are one and the same construct.

### MFT and RO Observed to Mutually Support Each Other

MFT researchers have challenged the empirical adequacy of RO constructs, worrying that RO has too narrowly focused on individual faith without sufficiently addressing its social dimensions. Such challenges to RO have been mounted mostly on theoretical grounds. Our findings suggest that MFT should reassess its criticisms of RO research. MFT and RO capture psychologically relevant differences within a large and demographically diverse sample of religious respondents. Moreover, MFT and RO offer mutually supporting approaches.

### Variation Observed Among Religious Adherents in MFT and RO

We found predictable associations between the variation of moral attitudes and the variation of religious orientations. We showed that not all religious people are cut from the same moral cloth. The moral variation of religion is evident from a large and demographically diverse sample of religious respondents from the same country. The relationship between religious cognition and moral cognition is a matter of intense empirical research [30]–[35]. Our findings contribute to the moral psychology of religion by showing that differences in moral foundations and religious orientations arise within a large and diverse sample of religious New Zealanders. Within-country variation – the premise of Religious orientations research – is a matter that MFT needs to take seriously.

### Questions For Future Research

Though New Zealand is a predominantly Christian country, a fact reflected in our predominantly sample of Christians, the religious diversity of New Zealand's Christian population has also been observed to be more extensive than in North America [36], and Christians in New Zealand have experienced steady declines in religious affiliations [37]. The relatively high levels of spiritual and supernatural beliefs in the country, however, suggest that the label “secular country” is misleading for New Zealand [38]. Given the size and demographic diversity of our sample, we are cautiously optimistic that our results generalize to religious adherents in New Zealand. Yet we cannot generalize to populations outside of New Zealand. Further studies are needed to assess whether the patterns we observed hold in other countries. Nevertheless, our study offers a theoretical proof that within-country variation in Moral Foundations may predict within-country variation in Religious Orientations.

A second related question is whether RO and MFT scales should be considered adequate to the task of addressing religious orientations and moral judgments worldwide. We would be surprised if they were adequate. Orthogonal research has found that RO scales may be unreliable for non-Christian religions [39]. By the same token, MFT is a fast-developing area of social psychological research. The fivefold categorization of classical MFT has recently been revised to include a sixth foundation – *Liberty* [13] p.61. The scales we used are unlikely to offer a comprehensive and ultimate set of categories by which to understand ways of valuing people, communities, and religions. If the moral psychology of religion is to remain intellectually vibrant, we expect future researchers will construct more satisfactory and informative scales.

A third question arising from our study is whether and how associations between MFT and RO translate into social behaviors. Unfortunately, such a question cannot be answered from our survey data. Future research must also consider whether any of the links between valuing religion and valuing other people affect social behaviors, whether such relationship vary by culture, and how these patterns change over time [40]–[42].

## Methods

### Ethics statement

The data reported in this study were collected as part of a larger longitudinal research project, The New Zealand Attitudes and Values Study. The longitudinal study was approved by The University of Auckland Human Participants Ethics Committee on 09-September-2009, reference number: 2009/336. Ethics approval for the study was re-approved by the University of Auckland Human Participants Ethics Committee on 17-February-2012 until 09-September-2015. Reference number: 6171. All participants gave written (online) consent. Participants provided consent when completing the questionnaire, in their own time, and in their own space. The Auckland Human Participants Ethics Committee approved this consent procedure.

### Participants and sampling procedure

We analyzed data from 1512 participants who identified as religious and who completed the 2012 mid-year wave of the New Zealand Attitudes and Values Study (NZAVS). The NZAVS is an annual longitudinal national probability sample of registered New Zealand voters, which was started in 2009 (see Hoverd 2008 for sampling details) [36]. The NZAVS 2012 mid-year wave was emailed to participants for whom email addresses were available (based on the full 2011 sample). A total of 4328 people completed the NZAVS 2012 online pre-survey of religion and morality. We limited our analyses to the 1512 respondents who identified as religious and completed the measures analyzed here (506 men, 982 women, 24 unreported). The mean age of people sampled was 52.13 years (SD  = 15.52). 11.8% of participants were Maori, 3.5% were of Pacific Nations ancestry, 5.6% were of Asian ancestry, and the remaining (79.1%) of the sample identified as either New Zealand European or with an “other” ethnic group. The majority of the sample (86.0%) identified with a Christian faith. The largest Christian denominational groups were: Anglican (16.4% of the sample), Catholic (18.6% of the sample), and Presbyterian (5.7% of the sample). A further 33.1% identified as Christian but offered no further definition (Christian NFD). The proportion of Christians in this religious sub-sample is broadly consistent with 2006 New Zealand census data, which estimated that 5.46% of the population identified with a non-Christian religious group (or roughly 9% of those who identify as religious). In an analysis of population trends, Hoverd (2008) suggested that the non-Christian proportion of religious people had increased since the New Zealand 2006 Census, which is consistent with the results from our sample, as 13.4% of religious respondents identified with a non-Christian faith [43].

### Measures

Moral Foundations were assessed using the 30-item Moral Foundations Questionnaire (MFQ-30). The MFQ-30 is available online (www.MoralFoundations.org, accessed September 3, 2013) and was developed by Graham and colleagues [17]. The scale contains six items for each of the Five Moral Foundations: Care/harm, Fairness/cheating, Loyalty/betrayal, Authority/Subversion, and Sanctity/degradation. Of the six-item scales for each of the five moral foundations, there are three items assessing the extent to which people use the various foundations when deciding whether something is right or wrong. The remaining three items relate to attitudes. The three measures assessed for each of these two item formats for each foundation are given equal weighting in determining overall scale scores.

To assess the criteria used to judge right or wrong, participants were asked: “when you decide whether something is right or wrong, to what extent are the following considerations relevant to your thinking?” Example items evaluating the use of different moral foundations when deciding matters of right and wrong are: Care/Harm:”Whether or not someone suffered emotionally;” Fairness/Cheating: “Whether or not some people were treated differently than others;” Loyalty/Betrayal: “Whether or not someone's action showed love for his or her country;” Authority/Subversion: “Whether or not someone showed a lack of respect for authority;” and Sanctity/Degradation: “Whether or not someone violated standards of purity and decency.” These items were rated on a scale from 1 (not at all relevant) to 6 (extremely relevant).

To assess moral attitudes, participants were given the following instructions: “Please read the following sentences and indicate your agreement or disagreement.” Example items assessing attitudes toward the different moral domains are: Care/harm: “Compassion for those who are suffering is the most crucial virtue;” Fairness/cheating: “When the government makes laws, the number one principle should be ensuring that everyone is treated fairly;” Loyalty/betrayal: “I am proud of my country's history;” Authority/Respect: “Respect for authority is something all children need to learn;” and Sanctity/degradation: “People should not do things that are disgusting, even if no one is harmed.” These items were rated on a scale from 1 (strongly disagree) to 6 (strongly agree).

Religious affiliation was evaluated by asking participants: “Do you identify with a religion and/or spiritual group?” (yes/no). Those who answered “yes” were then asked to complete the open-ended question, “If yes, then what religion/spiritual group?” After being presented with this question, those who indicated that they were religious were directed to an additional page in the on-line questionnaire that contained items that assessed their religious orientation and religious beliefs. All religious orientation items were rated on a scale from 1 (strongly disagree) to 7 (strongly agree).

Quest orientation was assessed using three items from the scale developed by Batson and Schoenrade [44]. The items used were: “I am constantly questioning my religious beliefs,” “There are many religious issues on which my views are still changing” and “As I grow and change I expect my religion also to grow and change”.

Intrinsic orientation was assessed using three items from the scale also employed by Batson and Schoenrade [44]: “My religious beliefs are what really lie behind my whole approach to life', “I try hard to carry my religion over into all my other dealings in life;” and “It is important for me to spend periods of time in private thought and meditation”.

Extrinsic-Personal orientation was assessed using three items from the scale also employed by Batson and Schoenrade [44]: “The purpose of prayer is to gain relief and protection;” “What religion offers me most is comfort when sorrows and misfortune strike,” and “The purpose of prayer is to secure a happy and peaceful life”.

Extrinsic-Social orientation was assessed using three items from the scale employed by Gorsuch and McPherson [45]: “I go to Church because it helps me to make friends', 'I go to Church mostly to spend time with my friends;” and “I go to church mainly because I enjoy seeing the people I know there”.

Bivariate correlations and descriptive statistics for scale mean scores are presented in **Table 1**.

**Table 1:** Bivariate correlations and descriptive statistics for scale mean scores.

	1	2	3	4	5	6	7	8	9
**1. Harm/Care**									
**2. Fairness/Reciprocity**	.591*								
**3. Ingroup/Loyalty**	.378*	.384*							
**4. Authority/Respect**	.236*	.228*	.596*						
**5. Purity/Sanctity**	.272*	.263*	.473*	.590*					
**6. Quest Orientation**	.049	.105*	−.095*	−.194*	−.155*				
**7. Intrinsic Orientation**	.180*	.150*	.092*	.097*	.368*	.075*			
**8. Extrinsic-Personal Orientation**	.241*	.253*	.330*	.283*	.245*	.080*	.171*		
**9. Extrinsic-Social Orientation**	.017	.062	.123*	.075*	.099*	.191*	.115*	.200*	

* **p<.01, N = 1188.**
